# Screening for Q Fever during Other Bacterial Endocarditis in Endemic Areas: Our Experience with Three Patients

**DOI:** 10.1155/2019/9890659

**Published:** 2019-07-08

**Authors:** Said Younis, Michal Stein, Sharon Reisfeld

**Affiliations:** ^1^Internal Medicine Department D, Hillel Yaffe Medical Center, Hadera, Israel; ^2^Ruth & Bruce Rappaport Faculty of Medicine, Technion, Haifa, Israel; ^3^Infectious Diseases Unit, Hillel Yaffe Medical Center, Hadera, Israel

## Abstract

Endocarditis is not a usual manifestation of acute Q fever. There is an ongoing debate about the need to screen patients for valvular diseases after acute Q fever. We present, for the first time, three patients with bacterial endocarditis from different aetiologies and a simultaneous diagnosis of acute Q fever. All were treated with prolonged antimicrobial treatment, and none of them developed a persistent Q infection. We suggest screening patients with endocarditis from other aetiologies to Q fever.

## 1. Introduction

*Coxiella burnetii* is the aetiology of 1–8% of bacterial endocarditis cases. Endocarditis is the most common form of persistent Q fever worldwide, while the manifestations of acute Q fever are asymptomatic infection, flu-like symptoms, pneumonia, or hepatitis, but not endocarditis [1]. Cases of coinfections of persistent Q infection and other bacteria as the causes of endocarditis were described previously [2, 3], but not coinfections of acute Q fever and other bacterial endocarditis.

## 2. Case Presentation

A 58-year-old healthy male was admitted to the internal medicine department due to weakness, fever, and night sweats for two months. On physical examination, a systolic heart murmur 3/6 was heard over the left sternal border. Abnormal laboratory findings included leukocytosis of 14,000 per microliter, hemoglobin of 9.9 gr/dL, and C-reactive protein (CRP) of 79 mg/L. Five sets of blood cultures were positive for *Streptococcus cristatus*, and transesophageal echocardiography (TEE) revealed severe aortic regurgitation with a large vegetation and moderate mitral regurgitation. The patient was treated with intravenous ceftriaxone and gentamicin and was referred to an aortic valve replacement, from which he recovered without complications. An immunofluorescent assay (IFA) for Q fever (methods described by Siegman-Igra et al. [4]) was positive for acute infection (phase II IgM = 100; phase II IgG = 400), and due to his risk factors for a persistent infection, he was treated with doxycycline and hydroxychloroquine, for a year, without adverse events or progression to a persistent infection. Follow-up serologic test results are given in Table 1.

Two similar patients were treated in our hospital. The first was a 72-year-old male with a prosthetic aortic valve, who was admitted due to one month of fever, weight loss, and weakness. He had splenomegaly and a purpuric rash in both legs, mild pancytopenia, and blood cultures that grew *Enterococcus faecalis* (three sets). TEE was unremarkable, and the patient was treated with intravenous ampicillin and gentamicin for six weeks with a good clinical recovery. The other patient was a 62-year-old male, with a prosthetic mitral valve, who was admitted due to four days of transient diplopia and fever. He had unremarkable physical examination, brain tomography, and TEE. He had elevated CRP of 109 mg/L and mild leukocytosis of 12,500 per microliter. *Streptococcus gordonii* grew in 9 sets of blood cultures, and he was treated with intravenous penicillin and gentamicin for 6 weeks.

Both of these patients had a 4-fold increase of phase II IgG that confirmed a diagnosis of acute Q fever, and they were treated with doxycycline and hydroxychloroquine for 4 and 12 months, respectively, and both had no clinical or laboratory signs of a persistent infection.

## 3. Discussion

Diagnosis of acute Q fever is based on serology, and the infection is asymptomatic in half of the cases [1]. Our patients definitely had acute Q infection, but the exact timing of the infection is less clear. We think that our patients have been exposed to the pathogen, with an asymptomatic or mild disease rather than a true coinfection. Since molecular tests from infected valves were not available, this remains a speculation.

Our patients had risk factors for recurrent endocarditis and were offered a prolonged antimicrobial treatment to reduce the risk for a persistent infection [5]. None of them progressed to such an infection. Should clinicians look for *Coxiella burnetii* in all patients with risk factors for a persistent infection, even when alternative diagnoses exist? This issue was not investigated in valvulopathies, but screening patients who are at high risk for a persistent vascular infection has been described previously and revealed 17% seropositive patients in the Netherlands [6]. Further studies are needed to establish an evidence-based policy.

## Figures and Tables

**Table 1:** Clinical, bacteriological, serological, and echocardiographic characteristics of three patients with bacterial endocarditis.

Patient	Previous risk factors	Results of blood cultures	Pathological echocardiographic findings	Surgery	First serology for Q fever	Duration of prophylaxis	Follow-up serology for Q fever (6–12 months from diagnosis)	Follow-up serology for Q fever (3–6 months from end of therapy)
1	None	*Streptococcus cristatus*	Severe aortic regurgitation and a large vegetation	Aortic valve replacement	IgM II-100 IgG II-400 IgM I-negative IgG I-200	12 months	IgM II-negative IgG II-200 IgM I-negative IgG I-negative	NA

2	Aortic valve replacement and aortic composite graft	*Enterococcus faecalis*	None	NA	IgM II-negative IgG II-400 IgM I-negative IgG I-100	4 months (stopped due to side effects: hyperpigmentation of the gingiva and calves)	IgM II-negative IgG II-100 IgM I-negative IgG I-negative	IgM II-negative IgG II-100 IgM I-negative IgG I-negative

3	Mitral valve replacement	*Streptococcus gordonii*	None	NA	IgM II-negative IgG II-200 IgM I-negative IgG I-negative	12 months	IgM II-negative IgG II-400 IgM I-negative IgG I-200	IgM II-negative IgG II-1600 IgM I-negative IgG I-400

## References

[1] Eldin C., Mélenotte C., Mediannikov O. (2017). From Q fever to *Coxiella burnetii* infection: a paradigm change. *Clinical Microbiology Reviews*.

[2] Yahav D., Kuznitz I., Reisfeld S., Eliakim-Raz N., Bishara J. (2015). Polymicrobial Q fever and enterococcal aortic prosthetic valve endocarditis with aortic root abscess. *Vector-Borne and Zoonotic Diseases*.

[3] Rovery C., Granel B., Casalta J.-P., Lepidi H., Habib G., Raoult D. (2009). Coinfection with *Coxiella burnetii* in infectious endocarditis. *Clinical Microbiology and Infection*.

[4] Siegman-Igra Y., Kaufman O., Keysary A., Rzotkiewicz S., Shalit I. (1997). Q fever endocarditis in Israel and a worldwide review. *Scandinavian Journal of Infectious Diseases*.

[5] Million M., Walter G., Thuny F., Habib G., Raoult D. (2013). Evolution from acute Q fever to endocarditis is associated with underlying valvulopathy and age and can be prevented by prolonged antibiotic treatment. *Clinical Infectious Diseases*.

[6] Hagenaars J. C. J. P., Wever P. C., van Petersen A. S. (2014). Estimated prevalence of chronic Q fever among *Coxiella burnetii* seropositive patients with an abdominal aortic/iliac aneurysm or aorto-iliac reconstruction after a large Dutch Q fever outbreak. *Journal of Infection*.

